# Artificial neural networks trained on simulated multispectral data for real-time imaging of skin microcirculatory blood oxygen saturation

**DOI:** 10.1117/1.JBO.29.S3.S33304

**Published:** 2024-07-10

**Authors:** Marcus Larsson, Maria Ewerlöf, E. Göran Salerud, Tomas Strömberg, Ingemar Fredriksson

**Affiliations:** a Linköping University, Department of Biomedical Engineering, Linköping, Sweden; b Linköping University, Department of Health, Medicine and Caring Sciences, Linköping, Sweden; c Perimed AB, Stockholm, Sweden

**Keywords:** multispectral imaging, blood oxygen saturation, microcirculation, artificial neural networks, Monte Carlo simulation

## Abstract

**Significance:**

Imaging blood oxygen saturation (${\mathrm{SO}}_{2}$) in the skin can be of clinical value when studying ischemic tissue. Emerging multispectral snapshot cameras enable real-time imaging but are limited by slow analysis when using inverse Monte Carlo (MC), the gold standard for analyzing multispectral data. Using artificial neural networks (ANNs) facilitates a significantly faster analysis but requires a large amount of high-quality training data from a wide range of tissue types for a precise estimation of ${\mathrm{SO}}_{2}$.

**Aim:**

We aim to develop a framework for training ANNs that estimates ${\mathrm{SO}}_{2}$ in real time from multispectral data with a precision comparable to inverse MC.

**Approach:**

ANNs are trained using synthetic data from a model that includes MC simulations of light propagation in tissue and hardware characteristics. The model includes physiologically relevant variations in optical properties, unique sensor characteristics, variations in illumination spectrum, and detector noise. This approach enables a rapid way of generating high-quality training data that covers different tissue types and skin pigmentation.

**Results:**

The ANN implementation analyzes an image in 0.11 s, which is at least 10,000 times faster than inverse MC. The hardware modeling is significantly improved by an in-house calibration of the sensor spectral response. An *in-vivo* example shows that inverse MC and ANN give almost identical ${\mathrm{SO}}_{2}$ values with a mean absolute deviation of 1.3%-units.

**Conclusions:**

ANN can replace inverse MC and enable real-time imaging of microcirculatory ${\mathrm{SO}}_{2}$ in the skin if detailed and precise modeling of both tissue and hardware is used when generating training data.

## 1 Introduction

Blood oxygen saturation (${\mathrm{SO}}_{2}$) is defined as the fraction of oxygen-saturated hemoglobin. Arterial ${\mathrm{SO}}_{2}$ can be monitored through pulse oximetry (PO) and provides information on how well the lungs oxygenate blood.^1^ Peripherally, the lack of significant blood pulsations makes PO insensitive to microvascular ${\mathrm{SO}}_{2}$. Instead, transcutaneous oximetry (${\mathrm{tcpO}}_{2}$) can be used to measure the locally available free oxygen that has diffused from the microvascular blood.^2^ The ${\mathrm{tcpO}}_{2}$ is a technique that only measures slow changes in one point. Spectroscopic optical techniques, including near-infrared spectroscopy^3^ (NIRS) and diffuse reflectance spectroscopy^4^^,^^5^ (DRS), allow for the fast and direct assessment of local ${\mathrm{SO}}_{2}$ levels in the microcirculation and smaller vessels. Due to a superficial sampling depth for DRS in the visible wavelength range, compared with NIRS, DRS mainly monitors capillary and upper dermis microvascular vessels, whereas NIRS includes deeper and larger vessels when measuring skin tissue.^6^

Microvascular networks are spatially heterogeneous and complex structures of arterioles, capillaries, and venules connecting the arterial and venous systems. Blood flow in the network varies temporally due to local regulatory and metabolic requirements of surrounding tissues and cells.^7^ Regulation involves adjusting organ perfusion to facilitate the oxygen exchange and carbon dioxide removal; the transport of hormones, nutrients, and drugs; and the immune response, among other functions. The local microvascular ${\mathrm{SO}}_{2}$ is hence a marker for tissue viability and could be used for studying different peripheral vascular diseases.^8^^–^^11^

Braverman et al.^12^ and Tenland et al.^13^ showed spatial heterogeneity in the microvasculature in healthy skin by studying variations in measured microcirculatory perfusion using single-point methods. The exact measurement position may, therefore, have a significant impact when determining skin microcirculatory disorders and regulation. Hence, imaging methods such as laser Doppler perfusion imaging,^14^ hyperspectral imaging (HSI), or multispectral imaging (MSI)^15^ may be preferred. Previous studies have shown that HSI and MSI can aid the diagnosis of diseases related to arterial occlusions such as peripheral arterial disease,^16^^,^^17^ diabetic foot,^18^ and wound diagnostics.^19^^,^^20^

Spectral images are often acquired by scanning through either the spectral or spatial domain, leading to long acquisition times and/or low resolution, hampering the widespread adoption of HSI and MSI.^21^ This includes push-broom setups,^6^^,^^22^ tunable Fabry–Pérot filters,^23^ or setups that cycle through wavelengths using filter wheels or multiple illumination bands.^24^ Using snap-shot sensors^25^^–^^28^ allows for much faster acquisition rates.

Analysis of HSI and MSI data is often slow when using advanced iterative algorithms such as an inverse diffusion model in a two-layer skin model^6^ or searches in multi-dimensional Monte Carlo (MC)-simulated reflectance look-up tables.^25^ Fast solutions, based on Beer–Lambert’s law in selected wavelength regions in single-layer skin models, have been proposed.^22^ MC simulations, however, give a more accurate solution to the photon transport equation in comparison with diffusion approximations and models based on Beer–Lambert’s law. The inverse MC method is considered the gold standard and thus should be the preferred choice for a precise analysis of DRS, HSI, and MSI data.

Recently, artificial neural networks (ANNs) trained on MC-simulated data from realistic multilayer skin models^23^^,^^29^ show that MC and ANN can be combined for a fast analysis of DRS and HSI data. Ewerlöf et al.^26^ also showed how ANN trained on *in-vivo* data from a reference device can be used for a precise and fast analysis of MSI snapshot images. This approach circumvents the need for handling the complex spectral response found in snapshot sensors, characterized by broad spectral bands or bands with multiple peaks. Unfortunately, it introduces the need for acquiring training data from a large representative population at different ${\mathrm{SO}}_{2}$ levels for every MSI snapshot camera as the sensor characteristics differ between each unique sample.

This study aims to develop a framework for training an ANN to estimate ${\mathrm{SO}}_{2}$ from snapshot MSI data with a precision comparable to inverse MC. The framework includes developing a model that closely mimics both tissue reflectance and instrumental characteristics for the generation of synthetic high-quality training data. An integral part of modeling is to account for variations in model characteristics to enable robust ${\mathrm{SO}}_{2}$ estimations insensitive to, e.g., tissue melanin, tissue scattering, instrumental noise, and instrumental drift, allowing for accurate real-time imaging of microcirculatory ${\mathrm{SO}}_{2}$.

## 2 Material and Methods

All images were acquired using an MSI snapshot camera (MQ022HG-IM-SM4X4-VIS, XIMEA GmbH, Münster, Germany) with 16 different optical bands in the 470 to 650 nm wavelength range. The camera had a complementary metal-oxide semiconductor (CMOS) imaging sensor with a native resolution of 2048×1088  pixels, where each pixel had a Fabry–Pérot bandpass filter deposited on top of it. The 16 unique bandpass filters were arranged in a recurring 4×4 mosaic pattern, resulting in an MSI hypercube (16×512×272). The camera was connected to a computer via USB3, allowing for a maximal theoretical frame rate of 170 fps. In our study, the actual frame rate was limited to 4.2 fps due to the chosen exposure time and on-the-fly averaging of multiple consecutive images.

The camera was equipped with a 16-mm c-mount lens (TECHSPEC, Edmund Optics, Barrington, New Jersey, United States) with the aperture size set to f/1.6. A long-pass (LP) filter with a cut-off wavelength of 470 nm (LP470 stablEDGE, Midwest Optical Systems, Palatine, Illinois, United States) was attached to the lens to suppress the dominant blue peak found in fluorescent white light-emitting diodes (LEDs). The spectral response for each of the 16 bands is influenced by, e.g., the attached optics and lens aperture size. Therefore, to complement the spectral responses supplied by the manufacturer, an in-house calibration was performed with the lens and LP filter attached. In addition, the spectral characteristics of the 8 W white LED ring light (R130, Smart Vision Lights, Norton Shores, Michigan, United States) and polarizing filters used in the *in-vivo* measurements were determined using a calibrated spectrometer (AvaSpec-2048L-USB2, calibrated using AvaLight-HAL-CAL-Mini; Avantes B.V., Apeldoorn, The Netherlands). The complete camera and LED setup was further characterized by repeated diffuse reflectance measurements from a white reference Spectralon target with a 99% nominal reflectance (Labsphere Inc., North Sutton, New Hampshire, United States). These measurements were used to describe temporal noise in the pixel intensity and to white-normalize all acquired *in-vivo* MSI data.

An optical model describing the camera system was combined with MC simulations of a tissue model describing the spectral reflectance of skin tissue. This combined model was used both in an inverse MC algorithm and for generating training data for an ANN algorithm trained to assess blood ${\mathrm{SO}}_{2}$ in skin tissue. The inverse MC and ANN algorithms were evaluated on *in-vivo* measured data from a healthy subject during an occlusion-release provocation.

### 2.1 Characterization of Sensor Spectral Response

**Fig. 1:**
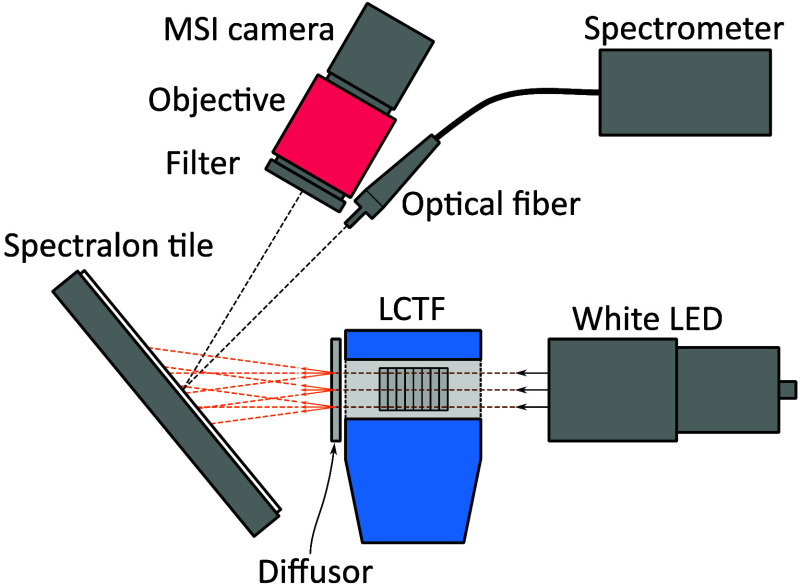
MSI camera calibration setup with a calibrated spectrometer and an LCTF-based tunable light source. The setup was used for quantifying the spectral response of the 16 different wavelength bands.

The spectral response of the 16 different wavelength bands in the MSI camera was characterized using the calibration setup in Fig. 1. The setup included a white LED spotlight (SXA30, Smart Vision Lights) mounted in front of a liquid crystal tunable filter (LCTF; VariSpec VIS-7, CRi Inc., McLean, Virginia, United States). A standard optical diffusor was placed after the LCTF to further mix and diffuse the filtered light. The light was then diffusely reflected by a white reference Spectralon tile and captured simultaneously by the MSI camera and an optical fiber connected to the calibrated spectrometer. The MSI camera included a 16-mm lens and LP470 filter.

The LCTF, with a 7 nm full width at half maximum (FWHM) transmission peak, was computer controllable, allowing for an automated scanning from 400 to 700 nm in steps of 1 nm. At each wavelength, MSI images and calibrated spectra were acquired. Dark MSI images and spectra were additionally recorded at every 10th wavelength.

Data were processed by first subtracting the dark level from the acquired MSI images and the high-resolution spectra acquired by the calibrated spectrometer. The high-resolution spectra were then smoothed with a 1 nm wide moving average filter, to avoid aliasing, and down-sampled to a 1 nm resolution between 400 and 700 nm. The MSI data were spatially averaged using a region of interest (ROI) that included all pixels with an intensity of more than 85% of the maximum intensity in the wavelength-averaged image. This resulted in a centrally positioned fixed-size ROI that included 11% of the sensor pixels.

The intensity detected by the MSI camera in the calibration setup is modeled as

$$I_{C}[n,m]=\overset{700}{\underset{\lambda =400}{\sum }}r_{n}(\lambda )R_{m}(\lambda ),$$ (1)

where n marks the spectral band for the MSI sensor, $r_{n}(\lambda )$ is the unknown response for each spectral band, and $R_{m}(\lambda )$ is the reflected irradiance as detected by the calibrated spectrometer while having the LCTF filter tuned to wavelength setting m. As a result of the 7 nm FWHM transmission peak of the LCTF, the spectral response is not given directly by the calibration dataset. Hence, the spectral response was estimated using a non-linear optimization algorithm to find the $r_{n}$ that minimizes the difference between the modeled and measured MSI intensities for all LCTF settings. Rapid changes in the estimated spectral response between consecutive wavelengths were additionally penalized to make the solution less sensitive to measurement noise.

The applied penalty function is given by

$$\chi_{r_{n}}=[\frac{I[n,m]}{{\left\langle I[n,m]\right\rangle }_{n,m}}-I_{C}[n,m],0.2\Delta r_{n}(\lambda )],$$ (2)

where I[n,m] is the intensity detected by the MSI camera in band n using LCTF wavelength m. The normalization with the average overall detected intensity ${\left\langle I[n,m]\right\rangle }_{n,m}$ makes the inverse solution insensitive to, e.g., overall lamp intensity, camera exposure time, and gain settings. No such normalization factor is needed for the modeled intensity $I_{C}[n,m]$ as this factor is instead handled by the overall magnitude of $r_{n}(\lambda )$. The additional term $\Delta r_{n}(\lambda )$ in the penalty function is given by

$$\Delta r_{n}(\lambda )=\frac{r_{n}(\lambda_{i+1})-r_{n}(\lambda_{i})}{0.5r_{n}(\lambda_{i})+0.5r_{n}(\lambda_{i+1})+{\left\langle r_{n}(\lambda )\right\rangle }_{\lambda }}.$$ (3)

The $\Delta r_{n}$ penalty term was designed as an amplitude-normalized derivative of the estimated sensor response. The normalization is equally balanced between the local and the global average amplitude. The local amplitude normalization improves spectral characteristics for low amplitudes, and the global amplitude normalization adds stability by avoiding zero division for a wavelength with zero response. The optimal response $r_{n}^{*}(\lambda )$ with the minimal penalty is found using a nonlinear least-square fitting algorithm (*lsqnonlin* in Matlab 2021a, The Mathworks Inc., Natick, Massachusetts, United States) that solved

$$\underset{r_{n}}{\min}\;{\left\|\chi_{r_{n}}\right\|}_{2}^{2}.$$ (4)

The optimal sensor response $r_{n}^{*}(\lambda )$ (a.k.a. *in-house* sensor response) and the sensor response supplied by the camera manufacturer were validated by comparing white-normalized measured and modeled MSI intensities from a tissue-like wide-band phantom. The validation data were acquired by replacing the LCTF with a skin-like transparent color filter (Roscolux #4615, Rosco Laboratories Inc., Stamford, Connecticut, United States). Spectral characteristics of the 470 nm LP filter were added to the spectral response in Eq. (1) when evaluating modeled intensities based on the sensor response data supplied by the manufacturer.

### 2.2 Modelling *In-Vivo* Detected MSI Intensities

**Fig. 2:**
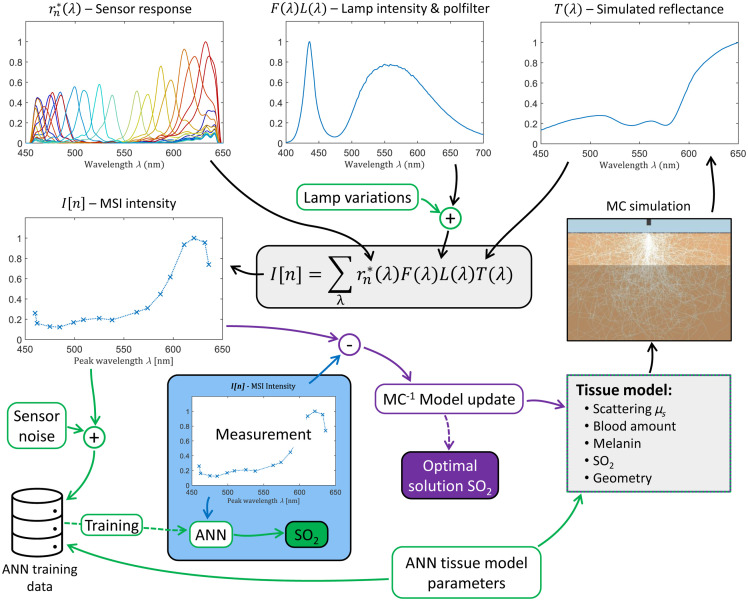
Schematic overview of how MSI intensities are modeled from the sensor response, lamp intensity, and MC-simulated reflectance using a two-layered skin tissue model. The modeled MSI intensities are used in the slow iterative inverse MC analysis (purple annotations) and the ANN training (green annotations), with variations and noise added, targeting estimation of the ${\mathrm{SO}}_{2}$ level. The blue box and lower blue arrow mark how MSI measurements are analyzed at high speed using a pre-trained ANN algorithm without needing any additional modeling of MSI intensities (in contrast to inverse MC).

Intensities detected by an MSI snapshot camera depend not only on the unique and complex response of each wavelength band but also on the LED irradiance, transmission of additional optical components, and reflectance of the target. Together, these effects contribute to the modeled detected intensity $I_{T}[n]$ in wavelength band n as outlined in the schematic overview in Fig. 2 and described by

$$I_{T}[n]=k_{n}\sum_{\lambda =400}^{700}r_{n}^{*}(\lambda )F(\lambda )L(\lambda )T(\lambda ),$$ (5)

where F(λ) is the spectral transmission of additional optical components (e.g., polarizing filter) not included in $r_{n}^{*}$, L(λ) is the LED emission spectrum, and T(λ) is the spectral reflectance of the target.^25^^,^^28^ The detected intensity in each pixel is also dependent on, e.g., local variations in the on-chip filter mosaic, setting of the lens, and analog-to-digital converters. The overall amplitude of these factors may vary spatially over the sensor and hence affect each band/pixel slightly differently. The local pixel-amplification effect is modeled by the coefficient $k_{n}$.

To effectively cancel out any influence from a $k_{n}$ that varies spatially over the imaging sensor, all MSI data were normalized with a hypercube measured on a white reference target. The white-normalized MSI spectra were also normalized with the local spectral-averaged intensity (i.e., average overall wavelength bands) to further reduce any dependency on, e.g., uneven emission intensity, target distance, and white calibration distance. For modeled intensities, this results in a normalized detected intensity that is calculated as

$$N[n]=\frac{I_{T}[n]\;}{I_{W}[n]}/{\left\langle \frac{I_{T}[n]\;}{I_{W}[n]}\right\rangle }_{n},$$ (6)

where ${\left\langle \ldots \right\rangle }_{n}$ denotes the average over all spectral bands and $I_{W}[n]$ is the MSI intensity from a white reference target modeled with T(λ)=1.

### 2.3 MC Model for Simulating Tissue Reflectance

The spectral response T(λ) of the tissue is modeled using MC simulations of a two-layer skin model. The model is a simplification of a three-layer model, used for DRS, that has been thoroughly described previously.^5^^,^^29^ The two-layer model consists of one epidermis layer with variable thickness $t_{\mathrm{epi}}$ and a variable tissue fraction of melanin $f_{\mathrm{mel}}$ and one dermis layer with an infinite thickness. The dermis layer consists of variable tissue fractions of blood $f_{\text{blood}}$, variable blood ${\mathrm{SO}}_{2}$ s, and variable vessel diameter D.

The reduced scattering coefficient is modeled to be equal for both layers according to

$$\mu_{s}^{\prime }(\lambda )=\alpha {\left(\frac{\lambda }{\lambda_{0}}\right)}^{-\beta },$$ (7)

where α and β are variable parameters and $\lambda_{0}=600\;\mathrm{nm}$. A Henyey–Greenstein scattering phase function with an anisotropy factor of 0.8 for all wavelengths was used in the simulations.

The absorption coefficient of the epidermis layer is calculated as

$$\mu_{a,\mathrm{epi}}(\lambda )=f_{\mathrm{mel}}k{\left(\frac{\lambda }{\lambda_{0}}\right)}^{\gamma_{\mathrm{mel}}},$$ (8)

where $k=39.0\;{\mathrm{mm}}^{-1}$, $\lambda_{0}=550\;\mathrm{nm}$, and $\gamma_{\mathrm{mel}}=-3$.^30^

The absorption coefficient of the blood in the dermis is calculated as

$$\mu_{a,\text{blood}}(\lambda )=s\mu_{a,\mathrm{oxy}}(\lambda )+(1-s)\mu_{a,\text{red}}(\lambda ),$$ (9)

where $\mu_{a,\mathrm{oxy}}$ is the absorption coefficient of blood with oxygenated hemoglobin and $\mu_{a,\text{red}}$ is the absorption coefficient of blood with reduced hemoglobin. Blood is modeled with a hematocrit of 43%, a hemoglobin concentration of 145  g/l blood, and a mean cell hemoglobin concentration of 345  g/l red blood cells (RBCs). The absorption spectrum for oxygenized blood is calculated from the absorption spectrum of oxygenized hemoglobin presented by Prahl,^31^ and that for reduced hemoglobin is calculated from the absorption spectrum of deoxygenized hemoglobin presented by Zijlstra et al.^32^

The absorption coefficient of the dermis is calculated as

$$\mu_{a,\mathrm{derm}}(\lambda )=f_{\text{blood}}c_{\mathrm{VD}}(\lambda )\mu_{a,\text{blood}}(\lambda ),$$ (10)

where $c_{\mathrm{VD}}$ is a vessel packaging effect factor, which is calculated as

$$c_{\mathrm{VD}}(\lambda )=\frac{1-\exp(D\mu_{a,\mathrm{derm}}(\lambda ))}{D\mu_{a,\mathrm{derm}}(\lambda )}.$$ (11)

White MC simulations were performed for various levels of epidermis thickness (2.5 to 490  μm) and scattering coefficient (1.0 to $90.5\;{\mathrm{mm}}^{-1}$). Each simulation ran until detecting 50 million photons. Various amounts of absorption were added in the post-processing using Beer–Lambert’s law and the pathlength for each photon in each layer. The results were stored in a four-dimensional grid where the detected intensity T could be interpolated for any values of $t_{\mathrm{epi}}$, $\mu_{s}^{\prime }$, $\mu_{a,\mathrm{epi}}$, and $\mu_{a,\mathrm{derm}}$.

### 2.4 Generating Data for ANN Training

The measured MSI spectra from tissue can be modeled by combining the MC tissue model and the MSI model, as outlined in the schematic overview in Fig. 2. However, these models do not fully include variations related to hardware imperfection. To synthesize realistic ANN training data, valid for all pixels on the sensor, such variations need to be added. We identified three different imperfections that significantly influence the MSI spectrum. This includes temporal drift in the LED emission spectrum, angular variations in the LED emission spectrum, and temporal noise-related variation in the detected intensity. Adding these three terms expands the modeled MSI spectrum in Eq. (5) to

$$I_{T,\mathrm{ANN}}[n]=k_{n}(1+\epsilon_{t,n})\overset{700}{\underset{\lambda =400}{\sum }}r_{n}^{*}(\lambda )F(\lambda )L_{\mathrm{ANN}}(\lambda )T(\lambda ),$$ (12)

where

$$L_{\mathrm{ANN}}(\lambda )=L(\lambda )(1+\delta_{t}(\lambda ))(1+\delta_{\theta }(\lambda )).$$ (13)

In these equations, L(λ) is the stable emission spectrum of the LED at a 0-deg emission angle, $\epsilon_{t,n}$ describes the temporal noise-related intensity fluctuation in each band n, $\delta_{t}(\lambda )$ relates to temporal drift in the emission spectrum, and $\delta_{\theta }(\lambda )$ adds emission-angle-dependent variations to the emission spectrum.

Changes over time in the factor $\delta_{t}(\lambda )$ were measured for the LED using a calibrated spectrometer at a 0 deg emission angle during a 60 min warm-up period. After this period, no significant drift was found, and the stable LED emission spectrum was determined as $L(\lambda )=L_{t=60\;\min}(\lambda )$. During the warm-up period, all $\delta_{t}(\lambda )$ were found to scale linearly to the maximum deviation as

$$\delta_{t,\max}(\lambda )=\frac{L_{t=0\;\min}(\lambda )}{L_{t=60\;\min}(\lambda )}-1,$$ (14)

where $L_{t=0\;\min}(\lambda )$ is the emission spectrum measured during the first minute of warm-up.

Variations in the factor $\delta_{\theta }(\lambda )$ were measured at emission angles between 0 and 22 deg, matching the maximal angle of view for the camera setup. All $\delta_{t}(\lambda )$ were found to scale linearly to the maximum deviation as

$$\delta_{\theta ,\max}(\lambda )=\frac{L_{\theta =22\;\deg}(\lambda )}{L_{\theta =0\;\deg}(\lambda )}-1,$$ (15)

where $L_{\theta =0\;\deg}(\lambda )$ is the stable emission spectrum measured at a 0 deg emission angle and $L_{\theta =22\;\deg}(\lambda )$ is the stable emission spectrum measured at a 22 deg emission angle.

**Fig. 3:**
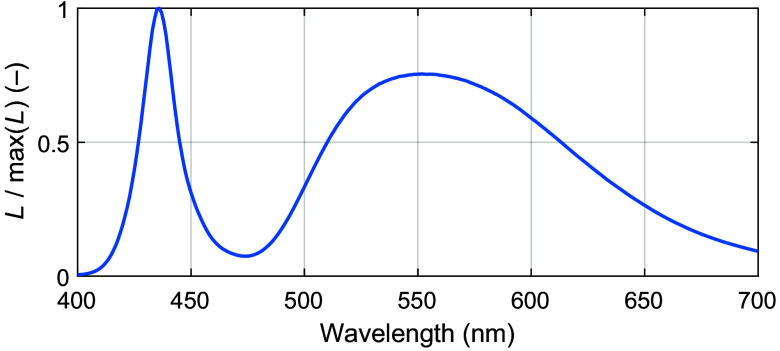
Normalized stable lamp emission spectrum measured at a 0 deg emission angle after a 60 min heat-up period.

With these linear relations, Eq. (13) is rewritten as

$$L_{\mathrm{ANN}}(\lambda )=L(\lambda )(1+q_{t}\delta_{t,\max}(\lambda ))(1+q_{\theta }\delta_{\theta ,\max}(\lambda )),$$ (16)

where $q_{t}\in [0,1]$ and $q_{\theta }\in [0,1]$. The spectral components for modeling the lamp emission spectrum in Eq. (16) are shown in Figs. 3 and. 4.

**Fig. 4:**
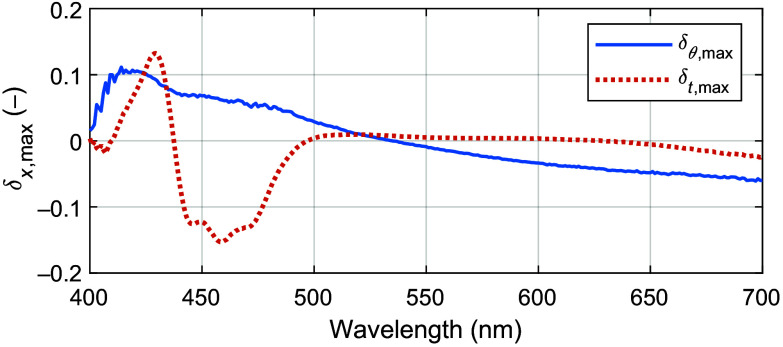
Maximal temporal ($\delta_{t,\max}(\lambda )$) and angular ($\delta_{\theta ,\max}(\lambda )$) variations in the lamp emission spectrum.

**Fig. 5:**
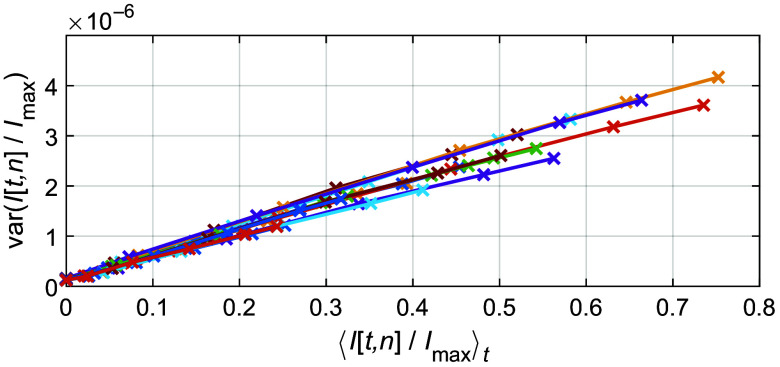
Variance of temporal detector noise for each of the 16 different bands. The intensity is normalized with the maximal detectable intensity $I_{\max}$ given by the camera analog-to-digital converter resolution.

The temporal detector noise $\epsilon_{t,n}$ depends on the light intensity quantified by each pixel. The noise is assumed to be Gaussian distributed with a variance that increases with intensity. Repeated white calibration measurements over time with the MSI camera, $I_{W}[t,n]$, at different LED light intensity levels, support the assumption of a Gaussian distribution. It also shows that the temporal noise variance increases linearly with intensity (Fig. 5), and hence, the detector noise is modeled by

$$\mathrm{var}(\epsilon_{t,n})=a_{n}+I[n]b_{n},$$ (17)

where $a_{n}$ and $b_{n}$ are coefficients determined from the calibration measurement and ${\left\langle \epsilon_{t,n}\right\rangle }_{t}=0$.

**Fig. 6:**
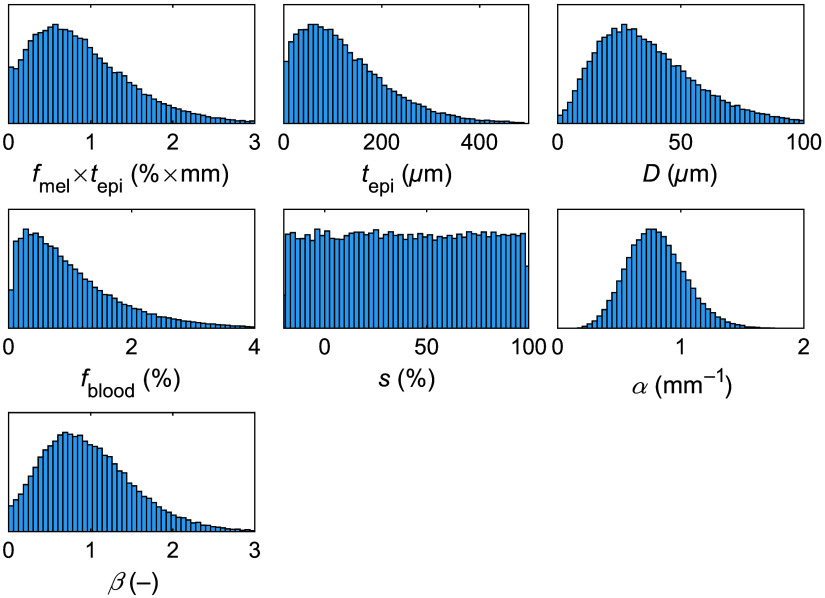
Distribution of the tissue model parameters for the ANN training set.

Having established a model that accounts for tissue and hardware variations, including noise, enables the synthetization of realistic MSI spectra that can be used for training an ANN to estimate blood ${\mathrm{SO}}_{2}$. The synthetization of a single MSI spectrum is done as follows:

1. generate an MC-simulated tissue reflectance spectrum T(λ) using a randomized set of tissue model parameters that comply with the expected parameter distribution found in real skin tissue (Fig. 6)*
2. generate an emission spectrum $L_{\mathrm{ANN}}(\lambda )$, where $q_{t}$ and $q_{\theta }$ are random numbers uniformly distributed at [0,1]
3. randomize an average detected MSI intensity $R_{I}$ from a normal distribution with a mean of half the maximal detectable intensity and a standard deviation of 20% of the maximal detectable intensity
4. calculate the intensity-normalized and scaled spectrum $I[n]=R_{I}I_{T,\mathrm{ANN}}[n]/{\left\langle I_{T,\mathrm{ANN}}[n]\right\rangle }_{n}$ assuming no temporal detector noise (i.e., $\epsilon_{t,n}=0$)
5. generate a detector noise factor $\epsilon_{t,n}$ with a variance according to Eq. (17) and Fig. 5.
6. generate a synthetic MSI spectrum according to Eq. (12) with the detector noise being added.
  * Please note that it is the amount of melanin, i.e., the product of the melanin fraction and the epidermis thickness, rather than the melanin fraction alone, that is randomized. The actual melanin fraction is then calculated from the melanin amount divided by the epidermis thickness.

The white normalization of measured MSI spectra was also accounted for in the models by normalizing the synthetic MSI spectra from skin tissue with synthetic white MSI spectra $I_{W,\mathrm{ANN}}[n]$. Synthetic white MSI spectra were generated as outlined above, except for using T(λ)=1 instead of an MC-generated tissue reflectance. Finally, the intensity normalization is calculated as

$$N_{\mathrm{ANN}}[n]=\frac{I_{T,\mathrm{ANN}}[n]}{I_{W,\mathrm{ANN}}[n]}/{\left\langle \frac{I_{T,\mathrm{ANN}}[n]}{I_{W,\mathrm{ANN}}[n]}\right\rangle }_{n},$$ (18)

where ${\left\langle \ldots \right\rangle }_{n}$ marks the average over all spectral bands. This effectively removes any dependency on the absolute magnitude of the MSI spectra.

### 2.5 Analyzing MSI Data Using Inverse MC and ANN

*In-vivo* MSI data were analyzed with inverse MC and ANN using both the spectral response supplied by the manufacturer and the optimal response $r_{n}^{*}(\lambda )$ measured as described above. The inverse MC analysis was done by modeling normalized MSI spectra using Eq. (6). The optimal solution with a minimal relative difference between the modeled and measured spectrum was found using a nonlinear least-square fitting algorithm (*lsqnonlin* in Matlab 2023a, The Mathworks Inc.) that searched the parameter space of $t_{\mathrm{epi}}$, $f_{\mathrm{mel}}$, $f_{\text{blood}}$, s, D, α, and β. For the first time-point in the *in-vivo* measurement, multiple start-points were used in the optimization to assure that the global optimum was found. For consecutive time points, the solution of the previous time point was used as a starting point.

ANN models with N[n] as input (i.e., 16 inputs), a single hidden layer containing either 5, 10, 15, or 20 nodes, and a single output targeting the blood ${\mathrm{SO}}_{2}$ were trained with synthetic MSI spectra generated as described in Eq. (18). The hidden layer had a hyperbolic tangent sigmoid transfer function, whereas the output layer had a linear transfer function. For each of the four network sizes, the training was repeated 10 times using a randomized initialization of the ANN model between training sessions. The training was done using the Levenberg–Marquardt backpropagation algorithm. In each training session, a single set of 50,000 synthetic MSI spectra was used and divided among training (70%), validation (15%), and testing (15%). The set of training data was MC generated using random parameters according to the tissue model described above.

### 2.6 *In-Vivo* Measurement Setup and Image Processing

As proof of principle, a single *in-vivo* measurement (male, age 48) was done targeting the dorsal side of the hand before (1 min), during (5 min), and after (4 min) an occlusion provocation of the middle finger. The subject was not suffering from any known microvascular disease, and he gave his informed consent before the experiment. The study design was approved by the Regional Ethical Review Board in Linköping, Sweden (D.no. 2018/282-31).

An average of eight consecutive images was done on the fly before saving each MSI dataset. This reduced image noise and data size while keeping an average frame rate of 4.2 fps. The *in-vivo* images were acquired using an exposure time of 20 ms. Interleaved dark images were collected every 20 s with the LED turned off.

The raw MSI images were preprocessed in the following way:

1. correct for ambient light effects by subtracting the nearest-in-time dark image
2. convert to a hypercube, using a weighted bilinear interpolation demosaic algorithm^33^
3. white-normalize using a calibration recording from a white Spectralon target; process the white hypercube as described above (steps 1 and 2)
4. intensity-normalize by dividing with the average spectral intensity in each hypercube pixel.

This processing replicates what is modeled in Eqs. (6) and (18), and consequently, it enables *in-vivo* data to be analyzed using either the trained ANNs or the inverse MC algorithm described above.

## 3 Results

**Fig. 7:**
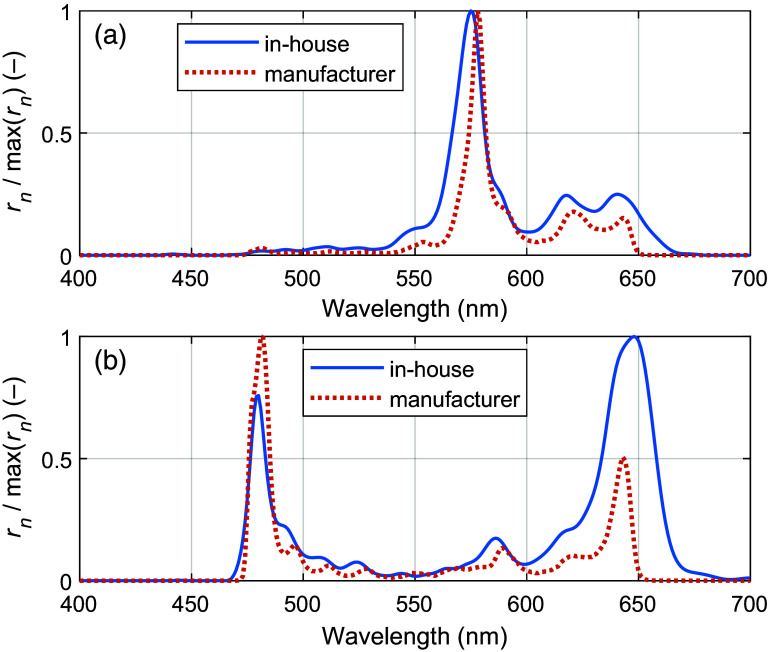
Representative examples of in-house and manufacturer-specified spectral responses for two different spectral bands. In panel (a), a typical widening and shift toward shorter wavelengths for the in-house spectral response is observed, and in panel (b), the worst matching case, a dual-peak characteristic in which the prominent peak changes position, is observed.

Data from the calibration setup show that the in-house optimal sensor response $r_{n}^{*}(\lambda )$ deviates from the manufacturer-supplied response by 3.4% to 9.6% (mean-absolute-error of max-normalized responses). Two representative examples of responses are shown in Fig. 7.

**Fig. 8:**
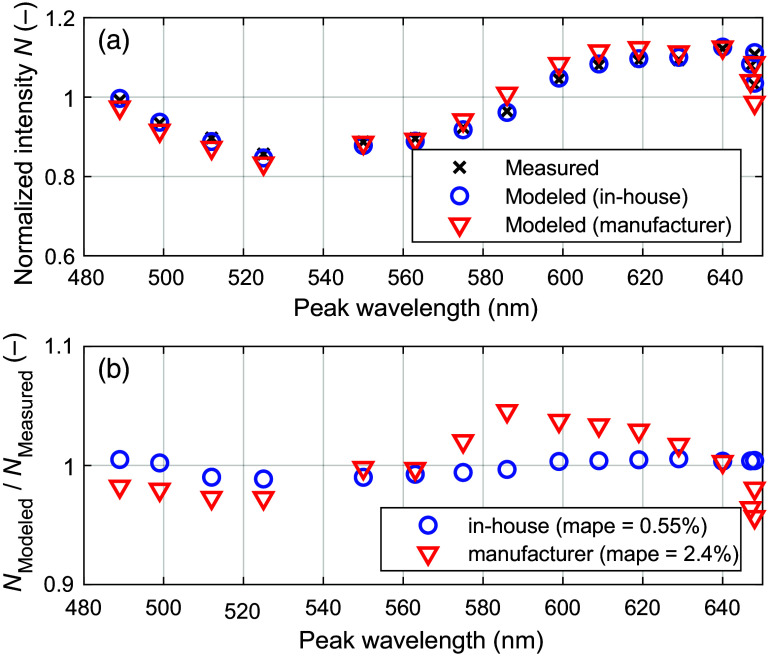
Phantom validation measurement comparing the in-house estimated spectral response with the one supplied by the manufacturer.

The validation measurement using a tissue-like color filter phantom shows that the in-house spectral responses can model the white-normalized measured intensities more accurately compared with the responses given by the manufacturer. The result, shown in Fig. 8, demonstrates a 4.3 times lower mean-absolute-percentage-error (mape) for the in-house spectral responses.

**Fig. 9:**
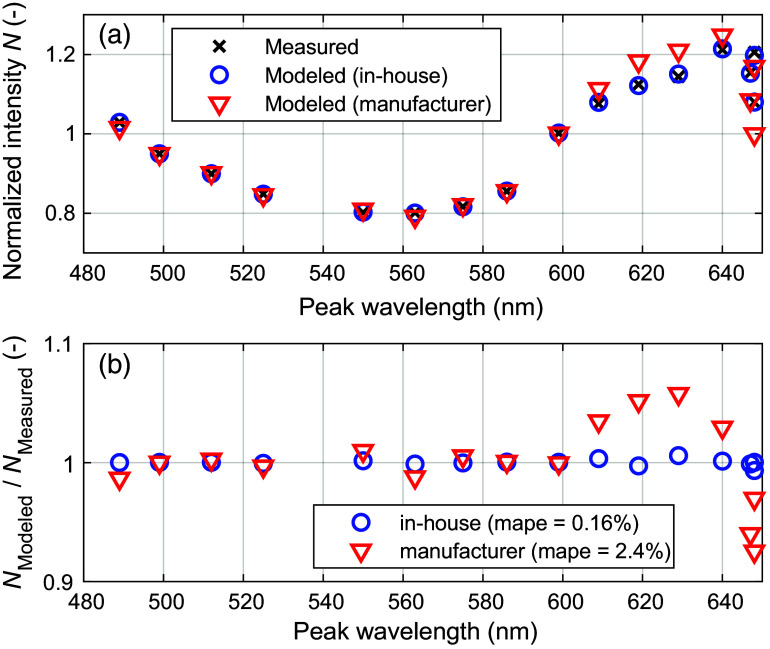
Example showing (a) measured and modeled normalized intensities and (b) the relative difference between measured and modeled intensities. The modeled data originate from an inverse MC fit to a single measured MSI spectrum during baseline. The measured data are ROI-averaged and analyzed with both the in-house and the manufacturer sensor responses.

**Fig. 10:**
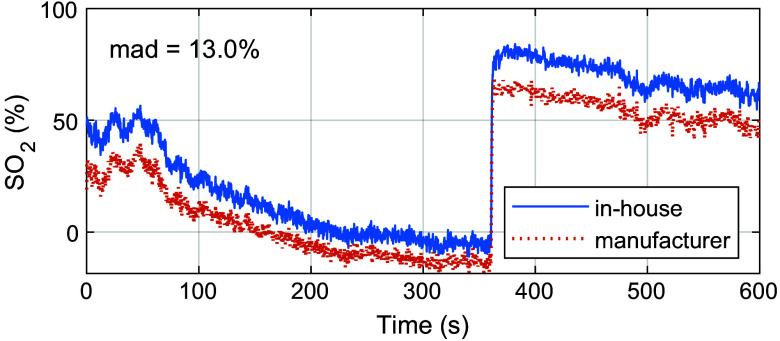
Inverse MC estimated blood ${\mathrm{SO}}_{2}$ during an occlusion-release provocation. ROI-averaged MSI data are analyzed using both the in-house and the manufacturer sensor responses.

The calibrated and normalized *in-vivo* MSI data from the finger occlusion experiment were analyzed by first selecting an ROI with a size of 5×5  pixels placed on the middle finger distal to the cuff position (Fig. 13). The ROI-averaged spectral data were then analyzed with inverse MC and the trained ANNs. The inverse MC analysis shows that the in-house spectral response yields significantly lower fitting errors (Fig. 9), with a time-averaged mape of 0.24% compared with a mape of 2.5% when using the manufacturer response. The time-resolved ROI data also show that the spectral response has a significant effect on the estimated level of blood ${\mathrm{SO}}_{2}$ (Fig. 10). The manufacturer response resulted in a consistently lower blood ${\mathrm{SO}}_{2}$ with a mean absolute difference ($\text{mad}={\left\langle |s_{\text{in-house}}-s_{\text{manufacturer}}|\right\rangle }_{t}$) of 13.0%, reaching an ${\mathrm{SO}}_{2}$ level of −13.7% during the last 10 s of occlusion. The equivalent level for the in-house response was −5.4%.

**Fig. 11:**
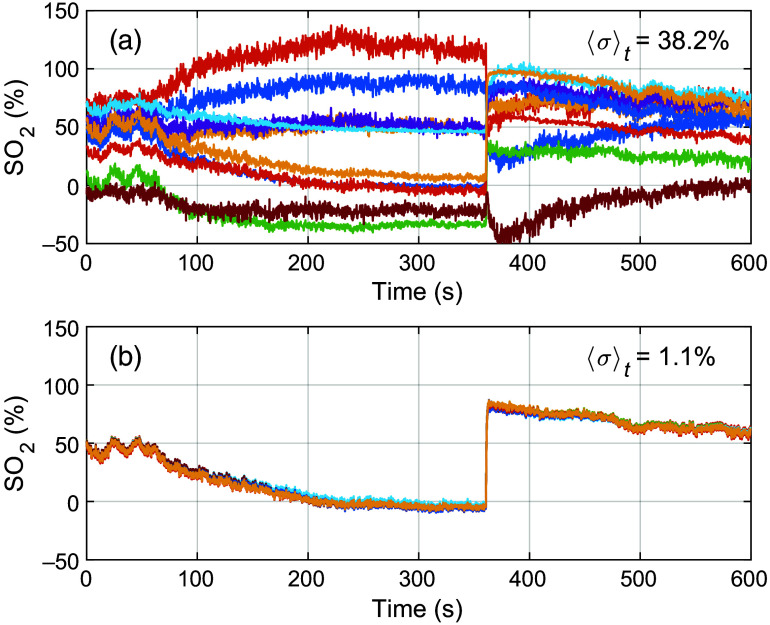
Example of ${\mathrm{SO}}_{2}$ estimations from 10 repeatedly trained networks, all having the same configuration with a hidden layer size of 15 nodes. The training data are generated with (a) the manufacturer response, with a time-averaged standard deviation of 38.2%-units, and (b) the in-house response, with a time-averaged standard deviation of 1.1%-units.

All ANNs from the repeated training using the in-house spectral response showed great consistency when applied to the *in-vivo* ROI data, with a time-averaged standard deviation in estimated blood ${\mathrm{SO}}_{2}$ of 1.1%-units to 1.8%-units for the different network sizes. The equivalent standard deviation for the ANNs trained with the manufacturer response was 5.9%-units to 60.0%-units, manifested as an almost random behavior with widely different time traces of estimated blood ${\mathrm{SO}}_{2}$ for the different repeatedly trained networks. The random behavior and standard deviation increased with the network size. Due to this inconsistency, no further meaningful comparison using the manufacturer response can be made as the results will strongly and randomly depend on which repetition of ANN training is selected. An example of ${\mathrm{SO}}_{2}$ estimations from 10 repeatedly trained networks, all having a hidden layer size of 15 nodes, is presented in Fig. 11(a) (manufacturer response) and Fig. 11(b) (in-house response).

**Fig. 12:**
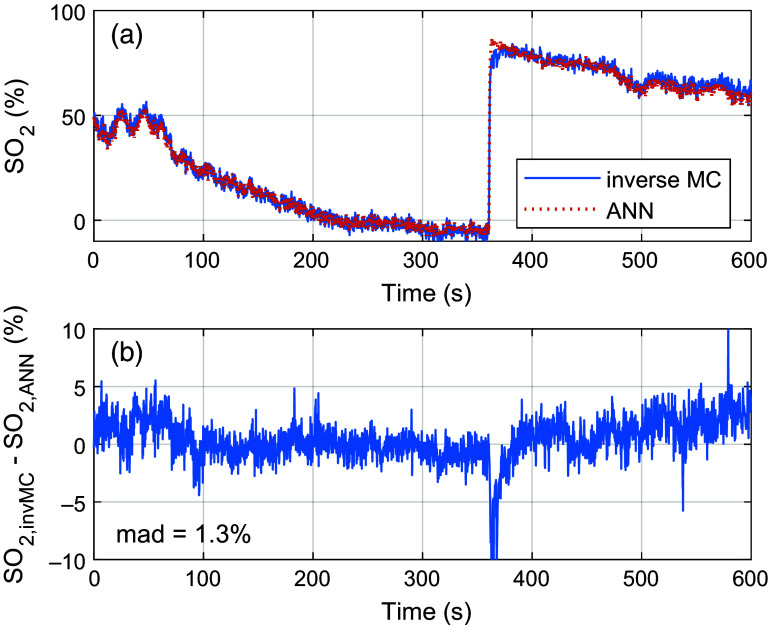
Panel (a) shows time traces of inverse MC- and ANN-estimated blood ${\mathrm{SO}}_{2}$ in the ROI. Panel (b) shows an absolute error between the two estimates.

ANN training performance (mean-square error evaluated with simulated test data) using the in-house response initially improved when increasing the number of nodes in the hidden layer. However, the improvement between a hidden layer size of 15 and 20 nodes was only marginal. Applying the ANN with the best test performance trained with the in-house response using a network size of 15 nodes demonstrates a close match to the time-trace of inverse MC estimated blood ${\mathrm{SO}}_{2}$ (Fig. 12). The mean absolute difference (mad) between inverse MC and ANN-estimated ${\mathrm{SO}}_{2}$ was 1.3%-units, and the coefficient of correlation R was 0.999. The only major performance difference between the two methods occurs during the initial reperfusion phase when ${\mathrm{SO}}_{2}$ rises quickly to its peak value. During this phase, the inverse MC algorithm fails to fully capture the rapid change, most likely due to stopping the inverse solver before finding the global optimum.

**Fig. 13:**
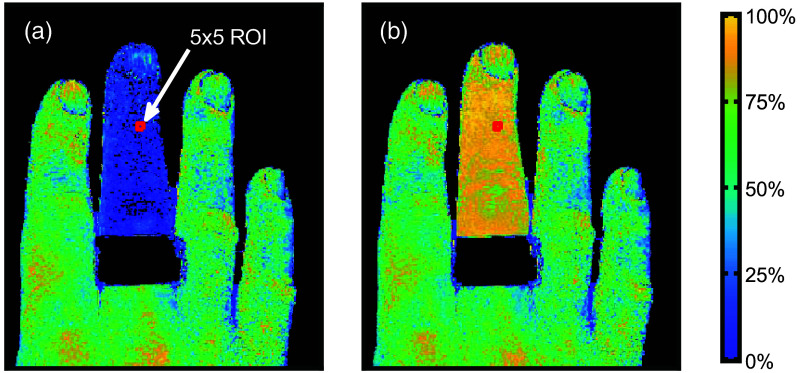
Examples of blood oxygen saturation still images from the ANN analysis showing an occluded (a) and reperfused (b) middle finger. The 5x5 pixel ROI, placed on the middle finger distal to the occlusion cuff, is marked in both still images.

ANN analysis of a single MSI cube, including preprocessing with dark correction, demosaicing, white normalization, and intensity normalization, took ∼0.11  s using a GPU implementation in Matlab R2021a (The MathWorks Inc.) and a GeForce GTX 1070 (Nvidia Corporation, Santa Clara, California, United States). The resulting blood ${\mathrm{SO}}_{2}$ images are shown in Video 1 and in Fig. 13, with representative still images of the occluded (at time 350 s) and a reperfused (at time 370 s) middle finger from the *in-vivo* provocation experiment. The images and video display the expected occlusion-release pattern with a close-to-zero ${\mathrm{SO}}_{2}$ at the end occlusion and ∼85% ${\mathrm{SO}}_{2}$ at the reperfusion peak. No apparent curvature-dependent effects can be seen, indicating that the demosaicing algorithm and the tissue model account for any such effects.

## 4 Discussion

We have shown that data from a snap-shot MSI camera with complex spectral characteristics can image ${\mathrm{SO}}_{2}$ in real time using an ANN trained on synthetic data. It is, however, critically important to apply a detailed and precise optical modeling of both the MSI camera system and the skin tissue.

The in-house spectral response, measured with optical components mounted to the MSI camera, and the response supplied by the manufacturer showed significant differences. In general, the in-house measured responses have peaks that are broader and shifted a few nanometers toward a shorter wavelength, as shown in Fig. 7. This effect is explained by the large lens aperture (f/1.6) used in this study, resulting in light impinging on the Fabry–Pérot filter deposited on top of the sensor at a range of different angles in contrast to the optimal orthogonal incidence.^34^ A smaller lens aperture could partly address this but would instead result in a lower signal-to-noise ratio. Preliminary measurements with a more recent version of the snapshot MSI camera indicate a better resemblance between the measured sensor response and the response specified by the manufacturer.

Evaluating differences in sensor response using a color phantom (Fig. 8) indicates that the manufacturer response is less accurate, suggesting that it is not fully valid for the lens and aperture setting used in our measurements. When applied to *in-vivo* data, the response difference is manifested as a large inverse MC fitting error when using the manufacturer response, as shown in Fig. 9. It is inevitable that this model error will lead to erroneous estimations of ${\mathrm{SO}}_{2}$. This can be seen in Fig. 10, for example, where ${\mathrm{SO}}_{2}$ assessed with inverse MC based on the manufacturer response deviates considerably from the one based on the in-house response.

Assessing ${\mathrm{SO}}_{2}$ with ANN results in standard deviations of up to 60%-units between ANNs that are repeatedly trained using the manufacturer response and random initialization. Using the in-house measured response drastically decreases this standard deviation to around 1%-units regardless of the network size. This shows that the ANN approach is much more sensitive to imperfections in the modeling of the sensor response. Previous studies have also shown that DRS training data must include realistic measurement noise and drift for accurate ANN results.^29^ For the MSI camera system, characterization and modeling of spatial and temporal variations in the light source emission spectrum and detector noise are required. The low standard deviation between outputs of repeatedly trained ANNs and the close match between inverse MC and ANN support the validity of these noise models.

Our results emphasize the need for an accurate calibration of the MSI camera response using the same optics, lens settings, and optical filters that are used in *in-vivo* measurements. The spectral response is a unique feature for each MSI sensor, which means that each individual camera must be characterized. Hahn et al.^35^ previously reported that this type of snapshot sensor may also have band-dependent variations in the sensor response across the detector area. Our work did not include any quantitative evaluation of this effect. The processed ${\mathrm{SO}}_{2}$ images from the *in-vivo* measurement showed no apparent major variation that could have contributed to this effect. This can be explained by the normalization with a white hyper cube, which effectively removes spatial variations in the response magnitude for each band.

Analysis of *in-vivo* measured data shows low fitting errors for the inverse MC algorithm and low standard deviations between repeatedly trained ANNs when using the in-house sensor response, as seen in Figs. 9 and 11. This strongly indicates that the applied tissue model is accurate and detailed enough to mimic the optical reflectance from skin tissue using MC simulations. The tissue model itself does not contain any limitation to the number of included absorbers or scatterers but is constrained by only including melanin and hemoglobin as absorbers, which seems sufficient for skin tissue, at least in the example presented in this work. Limitations in the model parameter space need, however, to be introduced when using the tissue model for inverse MC analysis as the algorithm relies on a pre-simulated set of simulated intensities. Similarly, when used for generating synthetic reflectance data for ANN training, all physiologically reasonable parameter variations must be covered. If not, the ANN algorithm is likely to perform poorly when used on tissue types that are not included in the model parameter space of the training data.

The overall behavior of ${\mathrm{SO}}_{2}$ in the *in-vivo* experiment (Fig. 12 and Video 1) follows the expected occlusion-reperfusion pattern when analyzing spectral data in the visible 500 to 600 nm wavelength range.^6^^,^^36^ The completely deoxygenized hemoglobin found after a prolonged occlusion can be explained by the superficial penetration depth for visible light, in which only blood in superficial microvascular vessels where oxygen is allowed to diffuse and metabolize in the surrounding tissue is sampled. This contrasts with techniques in which near-infrared wavelengths with a higher penetration depth are analyzed, resulting in elevated ${\mathrm{SO}}_{2}$ levels due to the sampling of blood in larger transport vessels.^6^

For transparency, we chose not to limit the estimated ${\mathrm{SO}}_{2}$ level to the 0% to 100% range. At the end occlusion, our *in-vivo* example shows an ${\mathrm{SO}}_{2}$ level of ∼−5% for both the inverse MC and ANN analyses in the selected 5×5 ROI. This is, of course, not physiologically possible. Increasing the ROI size to 25×25  pixels results in an ${\mathrm{SO}}_{2}$ level of −1.6% for the ANN analysis, indicating that some spatial noise is present, which, to some extent, causes the negative ${\mathrm{SO}}_{2}$ level. The negative ${\mathrm{SO}}_{2}$ values can also be explained by model errors, such as missing chromophores, and small calibration errors in, e.g., the white calibration of the MSI camera. The irradiance calibration is performed with a white reference target that has a very different reflectance compared with skin tissue. This can potentially introduce errors if the detected intensity response is not perfectly linear to the backscattered irradiance. With a well-known reference target mimicking the skin tissue, the effect of such non-linearities could be minimized. This is a strategy that has been successfully used in spatial-frequency domain imaging to minimize the impact of system imperfections.^37^

In a previous study with an optical fiber probe-based system, we showed that the type of MC model used in this study can be expanded to fully explain the diffuse reflectance spectra from a wide range of skin tissue types.^38^ In this work, however, we did not thoroughly explore what tissue model complexity is needed to produce algorithms capable of accurate modeling of MSI reflectance of different skin tissue types. Our *in-vivo* example has, however, uncovered (data not shown) that a single-layer tissue model or a model lacking melanin or vessel packaging effect cannot be fitted accurately to the measured data. Another limitation is the structure of the ANN, which has not been fully evaluated. The risk for overtraining certain parts or properties of the spectrum increases with a larger network size, and the limited input training data (16 spectral bands) should reduce the need for more complex or deep ANNs.^26^ We observe comparable results for ANNs trained with fewer nodes in the hidden layer. To further explore what tissue model complexity and parameter space is needed and how the ANN should be designed for a more generalized inverse algorithm, additional *in-vivo* measurements covering a range of different skin types are required. Adding a reference method to such a study would also aid in determining how well the technique works for different skin tissue types.

In a previous study,^26^ we used the same type of MSI camera and illumination setup but the ANNs were instead trained targeting ${\mathrm{SO}}_{2}$ values captured *in vivo* on the volar side of the forearm in 20 subjects (Fitzpatrick skin types I to III) using a reference probe-based technique. A leave-one-out analysis (i.e., training on 19 subjects and evaluating on the non-included subject) showed that the MSI ${\mathrm{SO}}_{2}$ estimation at end-occlusion was on par with (5%-units mean absolute error) the ${\mathrm{SO}}_{2}$ level measured with the reference technique. Those results show that ${\mathrm{SO}}_{2}$ can be estimated from MSI data for skin types I to III using an ANN-based approach if hardware and tissue can be modeled accurately. To further demonstrate the accuracy of the proposed method, a validation study using phantoms with a known ${\mathrm{SO}}_{2}$ level would be desirable.

Our ANN approach, in which ${\mathrm{SO}}_{2}$ is directly estimated, can analyze a single hypercube in ∼0.1  s. This is much faster than inverse MC, which typically takes up to 1 h to analyze a comparable-sized dataset.^25^ The 0.1 s analysis time demonstrates that our ANN approach, paired with a snapshot MSI camera, is fast enough for real-time imaging of ${\mathrm{SO}}_{2}$ dynamics at several frames per second. This frame rate allows us to capture most, if not all, physiological variations in ${\mathrm{SO}}_{2}$.

There are several clinical situations in which the utilization of HSI and MSI techniques is emerging. During free flap surgery, HSI at 16 to 28 h post-operatively utilizing a combination of tissue ${\mathrm{SO}}_{2}$ and a perfusion proxy index seemed to be superior to standard flap monitoring for predicting flap survival.^39^ In human forehead flaps, HSI mapping traced the expected decrease in ${\mathrm{SO}}_{2}$ during partial and full excision of the flap.^40^ In diabetes, the blood amount in the foot dermis and ${\mathrm{SO}}_{2}$ based on multispectral spatial frequency domain imaging could stratify subjects with varying severities of the disease.^41^ Furthermore, with the same technique, a low amount of blood in the superficial dermis and a high ${\mathrm{SO}}_{2}$ indicating poor oxygen transfer to tissue and a heterogenous light scattering were predictive of leg ulcer risk in a diabetic population.^42^

Other optical techniques for estimating ${\mathrm{SO}}_{2}$, e.g., near-infrared spectroscopy^43^ and HSI,^22^^,^^27^^,^^44^^,^^45^ often need to be calibrated using standard occlusion provocations or phantom experiments in which the ${\mathrm{SO}}_{2}$ level is known or measured with a reference technique. This is mainly due to the use of either simplistic inverse algorithms based on modified Beer–Lambert’s law expressions or pure empirical expressions in which only two or a few spectral components are analyzed. These algorithms are also typically sensitive to variations in tissue optical properties, including scattering and melanin content. The method presented in this work addresses these shortcomings through a thorough modeling of both instrumental effects and skin tissue variations.

## 5 Conclusion

Our results demonstrate that, with precise modeling of both hardware characteristics and tissue reflectance, ANN can estimate ${\mathrm{SO}}_{2}$ from MSI data with an accuracy comparable to inverse MC, a method often referred to as the gold standard for analyzing bio-optical signals. The use of ANN, trained on synthetically generated data, is computationally much more efficient than inverse MC and enables real-time ${\mathrm{SO}}_{2}$ imaging. The ANN approach is, however, more sensitive to imperfections in the modeling of the sensor response and requires noise and other hardware variations to be included when generating synthetic training data.

## 6 Appendix: Supplementary Video

**Video 1**: Example of blood ${\mathrm{SO}}_{2}$ imaging from the ANN analysis showing an occluded (60–360 s) and reperfused (>360 s) middle finger (MP4, 11.9 MB [URL: https://doi.org/10.1117/1.JBO.29.S3.S33304.s1]).

## Supplementary Material



## Data Availability

Please contact the authors regarding the availability of the code and data used in this study. Please note that the data management plan in the ethical approval prohibits us from sharing the *in-vivo* MSI data.
